# The Role of Infrared Thermography as a Non-Invasive Tool for the Detection of Lameness in Cattle

**DOI:** 10.3390/s150614513

**Published:** 2015-06-18

**Authors:** Maher Alsaaod, Allan L. Schaefer, Wolfgang Büscher, Adrian Steiner

**Affiliations:** 1 Clinic for Ruminants, Vetsuisse Faculty, University of Bern, Bern 3001, Switzerland; E-Mail: adrian.steiner@vetsuisse.unibe.ch; 2 Agriculture Forestry Centre, Department of AFNS, University of Alberta, Edmonton, AB T6G 2P5, Canada; E-Mail: alschaef@telus.net; 3 Livestock Technology Section, Institute for Agricultural Engineering, University of Bonn, Nussallee 5, Bonn D-53115, Germany; E-Mail: buescher@uni-bonn.de

**Keywords:** thermography, cattle, foot lesion, skin temperature, lameness

## Abstract

The use of infrared thermography for the identification of lameness in cattle has increased in recent years largely because of its non-invasive properties, ease of automation and continued cost reductions. Thermography can be used to identify and determine thermal abnormalities in animals by characterizing an increase or decrease in the surface temperature of their skin. The variation in superficial thermal patterns resulting from changes in blood flow in particular can be used to detect inflammation or injury associated with conditions such as foot lesions. Thermography has been used not only as a diagnostic tool, but also to evaluate routine farm management. Since 2000, 14 peer reviewed papers which discuss the assessment of thermography to identify and manage lameness in cattle have been published. There was a large difference in thermography performance in these reported studies. However, thermography was demonstrated to have utility for the detection of contralateral temperature difference and maximum foot temperature on areas of interest. Also apparent in these publications was that a controlled environment is an important issue that should be considered before image scanning.

## 1. Introduction

Lameness in cattle represents a major concern for the dairy industry globally both in terms of reduced production and compromised animal well-being. The majority of lameness in cattle is caused by claw abnormalities. Many studies report that diseases of the claw are associated with approximately 90% of all lameness incidents, with 76% to 84% of foot lesions occurring in the hind feet [1]. Traditionally, the diagnosis of foot lesions is often carried out by lifting the foot in a claw trimming chute during routine claw trimming or when the cow shows lameness [2,3,4]. Locomotion scoring is also a common subjective approach to assess lameness in cattle without lifting the foot [5,6,7]. However, this approach is time-consuming to use on the whole herd, particularly in larger herds and may not always be sensitive enough to detect foot lesions [8]. Moreover, foot lesions can be present without any sign of lameness, and so lameness does not appear until the lesion is severe [9,10]. Consequently, the early detection of a foot lesion can play an important role in reducing the negative impact of lameness, increases the treatment success, and is likely to be valuable in the prevention of further progression of pathology [11,12,13].

Infrared thermography is a noninvasive technique that measures emitted infrared radiation and displays the information as a pictorial representation, called a thermogram, of the surface temperature of an object [14,15]. Each pixel in the thermogram represents the measured surface temperature of an object. The information can be displayed in grey tones or as a colour scale. In a colour scale, the warmest areas are depicted as white or red, while the coolest areas appear blue or black [15,16]. Variations in the thermal (color) pattern reflect thermal gradients which represent changes in skin temperature due to underlying abnormalities.

One of the primary reasons to use infrared thermography to identify lameness is that if the lameness is due to inflammation there will be an obligatory thermal signature that can be identified as an image [17]. Thermography may not always provide specific pathology detail, however, it assists in defining the localization area of increased inflammation and/or injury. The inflammatory response is characterized by increases in the permeability of the blood vessels which results in increased blood flow that alters the heat pattern. The temperature of extremities and skin is largely dependent on the underlying circulation and tissue metabolism rate [18] and may further be influenced by individual differences among cows and environmental conditions [19,20,21]. Therefore, variation in superficial thermal patterns resulting from changes in blood flow [22] will alter the amount of radiated heat that may be easily identified by thermography and may relate to inflammation of tissues underlying that point or to changes in metabolic activity [23]. Thermography has been demonstrated as a useful tool to detect symmetry and asymmetry in surface temperature. Measuring the temperature differences in the affected area with healthy contralateral anatomical structures within an image allows the farmers to identify the significant differences among thermal images [15,24]. For example, asymmetry of 1 °C or more between two anatomically symmetrical regions is significant and may indicate a possible pathology in equine lameness [14]. As such, thermography may have great potential to assist diagnosis in bovine lameness. The evaluation of subtle temperature variation associated with inflammatory conditions could be a very important indicator to early detect inflammation associated with lameness.

The main benefit of using thermography is that non-invasive and accurate measurements of many subjects can be taken in rapid successions. However, a controlled environment or standardized operating procedure is often important to ensure the reliability of thermography imaging [15,21]. Combining thermography with other standard methods of lameness detection on-farm would improve the effectiveness and understanding of diagnostic competences of thermography.

This paper describes the clinical application of thermography to detect foot lesions and evaluates the current performance of thermography in the management of lameness in cattle. This paper starts with a section about its clinical application, followed by a description of the relevant internal and external factors, combining thermography with other farm information systems and finally the limitations/advantages of thermography.

## 2. Clinical Application

Table 1 lists peer-reviewed studies that used thermography for lameness management in cattle. Since 2000, 14 peer reviewed papers discussing the assessment of thermography to identify and manage lameness in cattle have been published. There was a large difference in thermography performance in these reported studies. In this section, the clinical application of thermography in bovine feet and joint disorders will be described and discussed.

### 2.1. Feet

**Figure 1:**
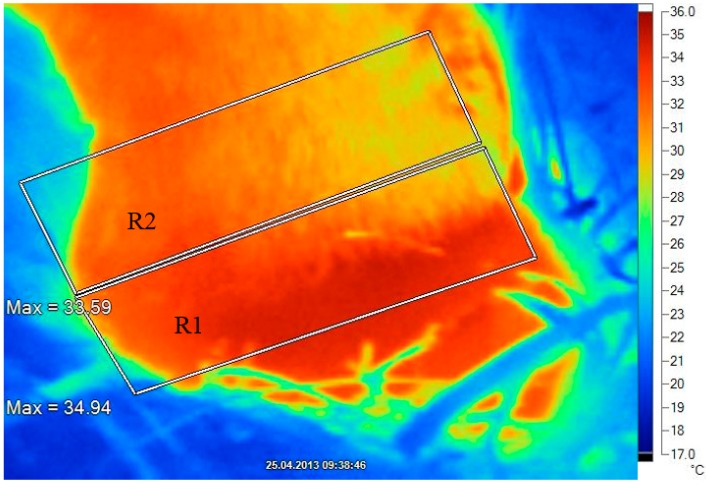
Infrared thermography image of the right rear foot (lateral aspect) as described by Alsaaod *et al.* [26,28]. R1 is the area of interest for measurement of the maximal temperature of the coronary band, defined as the hottest pixel measured by thermography in the junction between the skin and the horn of the claw; R2 is the area of interest for measurement of the maximal temperature of the skin, defined as the hottest pixel measured by thermography in the skin area, neighbouring R1 proximally.

Thermography equipment for use on farms can detect skin temperature differences with a precision of ±0.1 °C, and as such, temperature changes can often be seen before such changes are detectable by direct manual palpation. This fact is of relevance for the early diagnosis of foot lesions before the animal may show signs of pain, such as lameness. Consideration of the normal variations in thermal patterns of bovine feet that occur due to a number of factors, such as environment and an animal’s metabolism, is crucial to interpreting the changes in the thermal scans in clinical cases. In healthy feet, there is a temperature difference between front and hind feet, in which the hind feet are warmer than the front feet [25,26,27]. In the hind feet, the lateral claws are warmer [19,26], whereas in the front feet no difference between medial and lateral claws is apparent. Furthermore, the temperature of the coronary band is higher in early/mid-lactation cows compared with late lactation cows [19,21]. Foot surface temperature has mostly been taken at the coronary band and the surrounding skin region [19,21,26,28] (Figure 1). The reasons for this are: (1) The coronary band is a well vascularized area which optimally reflects the blood circulation of the claws, so that any inflammation or injury caused by foot lesion may be identified through a raised temperature at the coronary band and skin; (2) thermal scans of the coronary band and skin can be done without physical animal contact, quickly, and with minimal stress during examination; and (3) the coronary band can easily and clearly be identified by thermography, because the hair coat in this area is sparse and does not completely cover the coronary band. Hair can insulate and decrease the emission of infrared radiation to some degree [15]. Okada *et al.* [25] investigated the effect of the presence of hair on detecting object temperature at different ambient temperatures. For example, at room temperature, the detected temperature when hair was present, was significantly lower than when hair was absent (28.28 ± 0.36 and 33.9 ± 0.35 °C).

**Table 1:** Peer-reviewed published studies carried out from 2000 onward that used thermography for lameness detection in cattle.

Paper	Interest Area	View	Camera Type	^1^SE (%)	^2^SP (%)
Cockcroft *et al.* 2000 [26]	Metatarsophalangeal joint	Lateral, medial, plantar and dorsal	Hand-held IR imaging radiometer	-	-
Nikkhah *et al.* 2005 [19]	Coronary band and skin of lateral and medial claws	Dorsal	Infrared thermography	-	-
Rainwater-Lovett *et al.* 2009 [27]	bottom of the foot up to the top of claw	Dorsal	Infrared thermography	61.1	87.7
Gloster *et al.* 2011 [28]	around coronary band	Lateral, medial, plantar and dorsal	Infrared thermography	-	-
Alsaaod and Büscher, 2012 [21]	Coronary band and skin for lateral and medial claws	Dorsal	Infrared thermography	85.7 ^3^	55.9 ^3^
				80.0 ^4^	82.9 ^4^
Main *et al.* 2012 [29]	lateral and medial hind claws of hind feet	Plantar	Low cost Infrared thermometer	78	78
Stockes *et al.* 2012 [10]	Foot temperature	Plantar	Infrared thermography	80	73
Alsaaod *et al.* 2014 [30]	Coronary band and skin for lateral and medial claws	Lateral and medial aspect	Infrared thermography	89.1	66.6
Oikonomou *et al.* 2014 [31]	Sole area for lateral and medial claws	Solar	Infrared thermography		
Redaelli *et al.* 2014 [32]	Hind and fore limb	-	Infrared thermography	93 ^5^	50 ^5^
				38 ^6^	93 ^6^
Renn *et al.* 2014 [33]	Front and hind limbs	-	Handheld infrared thermography	-	-
Wilhelm *et al.* 2014 [34]	Sole area for lateral and medial claws	Solar	Infrared thermography		
Wood *et al.* 2014 [35]	Between heel bulbs and the accessory digits	Plantar	Infrared thermometer		
Alsaaod *et al.* 2015 [36]	Coronary band and skin for lateral and medial claws	Lateral and medial aspect	Infrared thermography		

^1^SE (sensitivity): True positive rate; ^2^SP (specificity): True negative rate; ^3^: Value at pre-trimming event; ^4^: Value at post-trimming event; ^5^: Value at hind feet; ^6^: Value at fore feet.

#### 2.1.1. Foot Lesions

There are several applications of thermography in cattle to assess foot and limb disorders. Whay *et al.* [30] examined the temperature of the claw at lateral aspects of the hind feet at the metatarsal joint, mid tarsus and the abaxial aspect of the lateral claw horn capsule. The results demonstrated an increased temperature in lame limbs as compared to sound limbs. Increased temperature at the coronary band measured by thermography corresponded with an increased incidence of sole ulcers, but not with incidences of underrun heels in the early to mid-lactation cows [19]. These authors concluded that measurement of foot temperature in early lactation may be useful for monitoring hoof health. Other studies measured the radiated temperature at the coronary band in cows without foot lesions, and demonstrated that there was no significant difference in surface temperature between left and right hind limbs but a significant difference in temperature when only one of these limbs was affected by a foot lesion [21,31]. Renn *et al.* [32] were able to classify 139 lame cows out of 142 when detecting temperatures over 27 °C, when one foot at least was affected with lameness. In their study, a high level of lame cows was recorded using thermography, suggesting this as an alternative objective diagnostic tool for lameness detection.

To assess the reliability of thermography as a diagnostic tool, an optimal cut-off value given by the thermography and occurrence of lesion detection (gold standard) should be established to determine the true positive rate (SE; sensitivity) and true negative rate (SP; specificity) between temperature thresholds. The sensitivity and specificity are codependent. With increasing the threshold of the test, the sensitivity will decrease and the specificity will increase. Therefore, the threshold of a test should be adjusted to determine the optimal sensitivity and specificity. For example, in populations with a high prevalence of foot diseases, it may more useful to select the healthy cases, in which high specificity of the test are required. The use of response operant condition (ROC) curves can assist in the identification of the optimal threshold.

The results of Alsaaod and Buscher [21] demonstrated an increase in the surface temperature of the coronary band of the affected foot as compared to the healthy contralateral foot: the threshold values established with the aim of detecting foot lesions were 0.64 °C at 3 days pre-trimming (SE = 85.7%, SP = 55.9%) and 1.09 °C at 3 days post-trimming (SE = 80.82%, SP = 82.9%). This suggests that each cow can serve as its own control for surface temperature variations. The authors concluded that these differences between healthy and lesion feet as measured by thermography may be useful for detection of hoof lesions in dairy cows without clinical inspection of the foot.

A recent study by Wood *et al.* [33] showed that thermography has the ability to detect the presence of an elevated temperature associated with foot lesions, although the technique did not allow differentiation among various lesions. They suggested a higher significant foot temperature at the point of lesion identification (estimated prediction value = 0.623 °C). Monitoring foot temperature over time showed a noticeable drop in average foot temperature six weeks following treatment, which may be associated with reduction of inflammation. These authors were able to identify the change of foot temperature six weeks’ prior to diagnosis of the lesion which suggested that thermography may be a useful tool in early detection of feet lesions in cattle [33].

Thermography has identified elevated temperature of the coronary band and temperature distribution patterns as possible detection methods for sole hemorrhage lesions [19,27]. Similarly, Stokes *et al.* [10] reported that the increasing maximum temperature of the plantar aspect of the hind feet was related to the presence of foot lesions including claw and digital dermatitis lesions. There was a difference between feet with and without foot pathologies. A cut-off temperature value of 27 °C was determined at 80% SE and 73% SP to define foot lesions of hind feet. Redaelli *et al.* [25] linked the thermographic measurements and veterinary foot diagnostics in healthy and lame cows. A sensitivity to identify lame cows of 93% and 50%, and a specificity of 38% and 93% were established for hind and fore feet, respectively.

Main *et al.* [34] assessed claw temperature, using a low-cost infrared thermometer immediately before claw trimming. The scanning was carried out from the plantar aspect of each hind foot and maximum cut off values were determined. The temperature of feet with lesions was higher compared to feet without lesions. A cut-off value of 25.25 °C was established to detect hoof lesions with a 72% SE and 73% SP. However, there was a large variation among the farms regarding the temperature of feet with or without lesions. Therefore the potential application of a low-cost infrared thermometer may be limited to monitoring claw temperatures of individual cows within herds. The mentioned studies were classified between lesion types upon a gross classification and not upon specific foot lesions. Further research was conducted to differentiate between types of lesions which had a significant impact on foot temperature. For sole lesions such as sole ulcers and hemorrhages, Nikkhah *et al.* [19] reported an increase in temperature at the coronary band in 16 lactating dairy cows in early/midlactation coinciding with increased incidence of sole hemorrhages compared with those in late lactation (mean ± SE; 25.4 ± 0.7 *vs.* 21.0 ± 1.5 °C), and suggested the use of thermography as a monitoring tool to detect sole lesions.

Thermography was demonstrated to be a reliable method to detect elevated temperature associated with Digital Dermatitis (DD) [26]. This study showed that thermography is a promising diagnostic tool in screening for the presence of DD in dairy cows by measuring the difference of maximum coronary band and skin temperatures between rear and front feet. The results of this study showed that feet with DD had significantly higher coronary band and skin temperatures than healthy feet. Using a threshold of 0.99 °C for the difference in coronary band and skin between rear and front feet, cows were detected with DD of the rear feet only with a sensitivity of 89.1% and a specificity of 66.6%. Furthermore, higher temperature was observed in feet with infectious status of DD (M2 and M4; M refers to Mortellaro) as compared to non-infectious DD-lesions. Early detection of DD is therefore likely to be valuable in preventing further progression and in providing effective treatment [35,36].

A possible explanation for these findings is that the inflammation response in the case of foot lesions is associated with increased blood flow and tissue metabolism rate leading to a localised increase in surface temperature that can be detected by thermography [18]. In this respect, multiple scanning images and comparisons between affected and healthy contralateral anatomical structures are useful in defining the consistency of an abnormality [14,21,26].

#### 2.1.2. Foot-and-Mouth Disease (FMD)

Viral infections typically result in the development of local or systematic inflammation characterized by increased blood flow. Previous work showed that thermography could be used as a screening tool for detecting animals suffering from foot-and-mouth disease (FMD) [37]. The results demonstrated that foot temperature (mainly around the coronary band) increased in animals infected with FMD. A cut-off value was established at 34.4 °C (SE = 61.1%, SP = 87.7) for detecting infected animals. It was concluded that thermography could be used as a rapid screening tool to help veterinarians to pre-select animals for further clinical examination and sampling. A further study reported a variation in foot temperature of healthy animals under various environmental conditions [38]. As a result, inflammatory conditions such as FMD could be detected by thermography, identifying abnormal surface temperature elevation.

#### 2.1.3. Routine Claw Management

Functional claw trimming in dairy cows is performed as a routine management procedure to prevent the development of claw disorders by maintaining the balance between lateral and medial claws and treating lesions if necessary. In cattle, thermography was used to evaluate the effect of routine claw trimming on claw temperature in cows housed in tie-stalls before and after claw trimming [28]. Analysing the maximum temperatures of coronary band and skin by thermography proved to be a useful technique to elucidate the effect of claw trimming on claw temperature. The difference in superficial temperature between the medial and lateral claws of the hind feet was observed to be decreased by routine claw trimming. Before claw trimming, the surface temperature of the lateral claws of the hind feet was significantly higher than that of medial claws, whereas such difference was not evident for the claws of the front feet. As a result, the attempt to equalize the weight bearing of the hind feet by preventive claw trimming is accompanied by a reduction of the temperature differences between the hind claws that is measurable with thermography.

Further to the above, Oikonomou *et al.* [39] reported the association between digital cushion thickness and sole temperature measured by thermography at the typical ulcer site of the lateral claw of hind feet. The thickness of the digital cushion measured by ultrasonography was associated with sole temperature in the immediate postpartum period and after claw trimming. The sole temperature was positively correlated with locomotion score and negatively with digital cushion thickness.

### 2.2. Joint Disorders

In a study from Cockcroft *et al.* [40] at the Queen’s Veterinary Hospital, Cambridge, thermography was used to diagnose septic arthritis of the metatarsophalangeal joint of a two-year-old Friesian heifer. By comparison with the healthy contralateral limb, the affected limb had a higher temperature at the level of the metatarsophalangeal joint in all lateral, medial, plantar and dorsal projections. The thermography identified the inflammation and provided supporting evidence for using thermography as a tool for localisation of the site of inflammation.

## 3. Internal and External Factors

Thermography is an extremely sensitive indicator of variations in heat patterns. For this reason thermoscans can also be easily influenced by extraneous factors that may create increased variation in results. For this reason, thermography images must be performed under controlled environmental conditions. Internal and external influences can alter the dynamics of blood flow and temperature regulation. Furthermore, it is likely that there may be individual animal variations that can change at different times of the day. One approach to minimizing individual animal’s variation is to report data of scanning images as a difference between affected and healthy contralateral anatomical structures to define the consistency of abnormality.

Factors which have been shown to affect the variability of infrared data include motion, environmental conditions such as ambient temperature, air flow, sunlight and humidity [41]. In addition, hair length and the distance between lens and claw may have an impact on the reliability of thermal images. Ideally, the ambient temperature should be within the thermal neutral zone and in a closed room without exposure to any direct sunlight or detectable airflow. A chosen model corrected for the effect of ambient temperature improves the accuracy for measuring temperature. Before scanning, the claws must be clean and dry. If the feet are dirty this has an effect both on surface temperature and emissivity value of the measured surface [27]. Hair has been shown to be an effective insulator by blocking heat emissions from the skin [14]. Therefore, hairless or clipped regions will appear warmer. Foot temperature measured by thermography is strongly and positively associated with ambient temperature [10,21,26,38,39]. Furthermore, motion immediately prior to a scanning session influenced the thermal results and produced false temperature elevations via increased peripheral circulation. Therefore, an acclimatization period of at least ten minutes is needed prior to an image being taken [38].

Appropriate calibration should be made on the camera to ensure the correct sensitivity and resolution of the image. In addition, using maximal temperature measured by thermography is often more revealing than the mean or minimum. A correlation was shown between the presence of foot lesions and maximum foot temperatures [10,26,30,37].

## 4. Combining Thermography with Other Information Systems

Claw disorders are associated with many changes occurring in a cow’s behavior. Therefore, combining thermography with other electronic methods of lameness detection (such as pedometer or 4-scale weighing platform) is potentially useful. Generally, a combination of different detection sensors has a better performance outcome than using a single sensor. Pedometer activity provides a non-invasive method to assess the locomotion behavior [42,43]. Combining behavior with thermography may be a helpful technique for preventing lameness. Under practical conditions in loose housing barns, the hygiene status of feet is much lower than in tie-stalls. As a consequence, it would likely be useful to combine thermography with “claw washer technologies” [44]. Another proposed method to improve the detection performance of thermography is combining the output of thermography with other non-sensor information like lameness history.

## 5. Advantages and Limitations of Infrared Imaging

A major advantage of thermography is the fact that it measures heat emissions and does not need direct physical contact with the surface measured, thus allowing the monitoring of temperature distribution non-invasively. Infrared is also more sensitive than palpation for detecting subtle temperature variations. This high sensitivity makes it useful in combination with other specific information (such as pedometer activity). Generally, thermography is best used in combination with other modalities rather than as a replacement for them. Thermography often reveals physiological changes before they appear as clinical signs, thus providing early detection and allowing for early treatment intervention [17]. Similar to other diagnostic methods, thermography becomes a most valuable tool when its abilities and limitations are known and recognized. Furthermore, thermography equipment costs are becoming lower every year and thus make this technology affordable for more farms to detect foot temperature in commercial practice and so allow for rapid screening. Further to the above, the use of a rapid and reliable non-invasive tool to screen large numbers of animals without any need for handling would allow a more efficient use of valuable and often limited labour resources. While thermography provides localization and physiological information, the definition of etiologies is a continuing work in progress.

## 6. Conclusions/Outlook

The use of thermography to improve the detection of lameness in cattle has gained interest. This is largely because it represents a non-invasive and rapid screening technique that can be used automatically within a long-term monitoring program. Thermography may not always reveal specific pathology detail, however, it assists in defining the localization area of inflammation and/or injury or increased metabolism or decreased heat (reduced blood flow). Thermography can detect skin temperature difference with a precision of ≤ ± 0.1 °C, which can occur before such changes can often be detected by direct manual palpation. That sensitivity assists with the diagnosis of foot lesions before the animal shows signs of pain, such as lameness. When evaluating a detection model using thermography, it is very important to consider the environmental conditions and animal individuality. Environmental factors, such as air temperature, dirt and debris and humidity can affect the thermographic scanning [41]. Moreover, it is often very important to consider the within-animal temperature difference rather than absolute values. Maximum temperature of the area of interest is often more revealing than the average values. An acclimatization period of at least 10 min is needed prior to an image being taken. For detection of lameness, the combination of washing and thermography may be optimal for the management of cows kept in free stalls (cubicles).
